# PPARgamma Deficiency Counteracts Thymic Senescence

**DOI:** 10.3389/fimmu.2017.01515

**Published:** 2017-11-06

**Authors:** David Ernszt, Krisztina Banfai, Zoltan Kellermayer, Attila Pap, Janet M. Lord, Judit E. Pongracz, Krisztian Kvell

**Affiliations:** ^1^Faculty of Pharmacy, Department of Pharmaceutical Biotechnology, University of Pecs, Pecs, Hungary; ^2^Szentagothai Research Center, University of Pecs, Pecs, Hungary; ^3^Faculty of Medicine, Department of Immunology and Biotechnology, University of Pecs, Pecs, Hungary; ^4^Faculty of Medicine, Department of Biochemistry and Molecular Biology, University of Debrecen, Debrecen, Hungary; ^5^College of Medical and Dental Sciences, Institute of Inflammation and Aging, University of Birmingham, Birmingham, United Kingdom

**Keywords:** PPARgamma, thymus, immunity, senescence, rejuvenation

## Abstract

Thymic senescence contributes to increased incidence of infection, cancer and autoimmunity at senior ages. This process manifests as adipose involution. As with other adipose tissues, thymic adipose involution is also controlled by PPARgamma. This is supported by observations reporting that systemic PPARgamma activation accelerates thymic adipose involution. Therefore, we hypothesized that decreased PPARgamma activity could prevent thymic adipose involution, although it may trigger metabolic adverse effects. We have confirmed that both human and murine thymic sections show marked staining for PPARgamma at senior ages. We have also tested the thymic lobes of PPARgamma haplo-insufficient and null mice. Supporting our working hypothesis both adult PPARgamma haplo-insufficient and null mice show delayed thymic senescence by thymus histology, thymocyte mouse T-cell recombination excision circle qPCR and peripheral blood naive T-cell ratio by flow-cytometry. Delayed senescence showed dose–response with respect to PPARgamma deficiency. Functional immune parameters were also evaluated at senior ages in PPARgamma haplo-insufficient mice (null mice do not reach senior ages due to metabolic adverse affects). As expected, sustained and elevated T-cell production conferred oral tolerance and enhanced vaccination efficiency in senior PPARgamma haplo-insufficient, but not in senior wild-type littermates according to ELISA IgG measurements. Of note, humans also show increased oral intolerance issues and decreased protection by vaccines at senior ages. Moreover, PPARgamma haplo-insufficiency also exists in human known as a rare disease (FPLD3) causing metabolic adverse effects, similar to the mouse. When compared to age- and metabolic disorder-matched other patient samples (FPLD2 not affecting PPARgamma activity), FPLD3 patients showed increased human Trec (hTrec) values by qPCR (within healthy human range) suggesting delayed thymic senescence, in accordance with mouse results and supporting our working hypothesis. In summary, our experiments prove that systemic decrease of PPARgamma activity prevents thymic senescence, albeit with metabolic drawbacks. However, thymic tissue-specific PPARgamma antagonism would likely solve the issue.

## Introduction

The peroxisome proliferator-activated receptor (PPAR) molecular family is widely studied (1–3). These nuclear receptor proteins possess transcription factor activities and influence multiple cellular events at the molecular level including adipocyte differentiation and metabolism. Among them, PPARgamma is of particular interest being expressed by all adipose tissue subtypes and being indispensable for adipose tissue development and for the homeostasis of physiological metabolism (4–7). As a consequence, in the mouse systemic loss of PPARgamma activity severely impairs glucose and lipid metabolism as characterized by others (8–10). In accordance, PPARgamma null mice are only viable if using conditional knockout strategy (11). Similar to the mouse above, in human PPARgamma haplo-insufficiency leads to the development of a rare metabolic condition known as familial partial lipodystrophy, type 3 (FPLD3, ORPHA 79083) also characterized by diabetes and dyslipidemia (12–15).

In mammals, systemic PPARgamma activity may be increased at multiple levels. Environmental factors including excessive caloric consumption or corticosteroid exposure increase PPARgamma activity systemically (16–18). Pharmacological systemic activation may be achieved through administration of thiazolidinediones previously used as part of oral antidiabetic treatment, but currently neglected due to adverse cardiovascular side effects (19, 20). Genetic engineering-based enhancement of PPARgamma activity in mouse models has also been performed (21). In every case, increased PPARgamma activity promotes adipose tissue development at multiple sites of the body.

Thymic aging is observed as adipose involution during which the functional thymus niche that normally supports T-cell production is gradually lost and replaced by adipose tissue (22). The process starts focally in childhood then spreads and accelerates with puberty due to hormonal changes (23). Diminishing T-cell production results in decreased availability of fresh naive T-cells (24). Consequences include increasing incidence of infection, cancer and autoimmunity observed at senior ages (25, 26). Thymic adipose involution appears to be PPARgamma-dependent: any condition that systemically enhances PPARgamma activity—either environmental, pharmacological, or genetic—accelerates thymic senescence or adipose involution with all its immunological consequences (27–32). However, the opposite phenomenon whether systemically decreased PPARgamma activity can ameliorate long-term functional immune parameters has barely been addressed (33, 34). For this reason, we have set out to characterize the effect of systemic genetic PPARgamma loss of function on long-term immune homeostasis in both mouse and human.

## Materials and Methods

### Human Thymus Samples

Formalin-fixed, paraffin-embedded (FFPE) human thymus samples from age groups 30–40, 50–60, and 70–80 years were obtained from the Department of Pathology (Faculty of Medicine, University of Pecs, Hungary.) Experiments involving human thymus samples were performed with the consent of the Regional and Local Ethics Committee of Clinical Centre, University of Pecs (ref. no.: 6331/2016) according to their guidelines. All subjects gave written informed consent in accordance with the Declaration of Helsinki.

### Human Immunohistochemistry

Human thymus lobes were fixed in paraformaldehyde (4% PFA in PBS) then paraffin embedded. 5 µm thick sections were stained using immunohistochemistry (35). First, the slides were rinsed in heated xylene and were washed with a descending series of alcohol to remove paraffin. After deparaffination the slides were rehydrated in distilled water and antigen retrieval was performed by heating the slides in Target Retrieval Solution (pH 6 DAKO) at 97°C for 20–30 min. Subsequently slides were washed in dH_2_O and endogenous peroxidase activity was blocked with 3% H_2_O_2_ containing TBS (pH 7.4) for 15 min. Then slides were washed three times with TBS containing Tween (0.05%, pH 7.4). Pre-blocking was carried out with 3% BSA in TBS for 20 min before overnight incubation with anti-PPARgamma (1:100, rabbit monoclonal antibody clone: C26H12 Cell Signalling Technology) primary antibody at 4°C. Following incubation slides were washed with TBS for three times then incubated with peroxidase conjugated secondary antibody (1:100, Polyclonal Goat Anti-Rabbit IgG, DAKO) for 90 min. Antibody labeling was visualized with the help of liquid DAB Substrate Chromogen System (DAKO). For nuclear counterstaining, hematoxylin staining was performed. Finally, slides were mounted with Faramount Aqueous Mounting Medium (DAKO). Histological evaluation was performed with the help of Panoramic MIDI digital slide scanner (3DHistech). Image analysis was performed using ImageJ software with IHC toolbox plug-in.

### Mouse Breeding and Maintenance

For certain experiments, we have used wild-type and PPARgamma heterozygous (haplo-insufficient) or PPARgamma null (KO) mice of C57BL/6J genetic background. The mice were age matched, and both genders were used for the investigation. The design to generate PPARgamma KO mice was described previously (11). Briefly, PPARgamma^+/−^/Sox2Cre^+^ male mice were crossed with PPARgamma fl/fl female mice to generate heterozygous PPARgamm^afl/−^/Sox2Cre^−^ and homozygous PPAR gammaΔ^fl/−^/Sox2Cre^+^ mice, wherein the floxed allele was recombined resulting a null allele. Mice were housed under minimal disease conditions in the Laboratory Animal Core Facility of University of Debrecen. Animal rooms were ventilated 15 times/h with filtered air, mice received autoclaved pellet diet (Altromin VRF1) and tap water *ad libitum*. The cages contained sterilized bedding. Room lightning was automated with 12 h light and 12 h dark periods. The room temperature was 21 ± 2°C, the relative humidity is between 30 and 60%. Senescent animals developed and aged normally, without any treatment. Permission to perform the described animal experiments was granted to the relevant utilities of the University of Pecs (ref. no.: BA02/2000-46/2016). Permission to generate PPARgamma GM mice was granted to the relevant utilities of the University of Debrecen (ref. no.: TMF/82-10/2015). Permission to perform experimental procedures with PPARgamma GM mice was granted to the relevant utilities of the University of Pecs (ref. no.: TMF/124-11/2017).

### Mouse Immunofluorescence

Immunofluorescent staining was performed on 8 µm cryosections of mouse thymus lobes as described previously (35). Briefly, the slides were fixed in cold acetone, then dried and blocked to prevent non-specific staining using 5% BSA in PBS for 20 min before staining with fluorochrome-conjugated or primary antibodies: anti-EpCAM1-FITC (1:100, rat monoclonal antibody clone: G8.8), anti-Ly51-PE (1:100, rat monoclonal antibody clone: 6C3, eBioscience), and anti-PPARgamma (rabbit monoclonal antibody clone: C26H12 Cell Signaling Technology). For secondary antibody, Alexa-555 conjugated a-rabbit goat IgG (1:200, Life Technologies) was used. In certain cases, DAPI (Life Technologies) nuclear counterstain was also applied. Sections were analyzed using a Nikon Eclipse Ti-U microscope equipped with a CCD camera (Andor Zyla 5.5) and NIS-Elements software. The medulla/cortex ratio was calculated using ImageJ software.

### Mouse Flow Cytometry

Thymocyte subsets and T-cell subpopulations in blood were investigated by flow-cytometry as published by others (36, 37). Thymocytes and PBMC were isolated from mice and labeled with fluorophore-conjugated antibodies in PBS-BSA (5% BSA diluted in PBS). In every case, 100,000 cells were stained for measurement. Incubation with antibodies was performed at 4°C for 60 min followed by a washing step. FACSCanto II flow-cytometer and FACSDiva software (Becton Dickinson) were used for analysis. In every case, 10,000 events (parent R1 morphological lymphocyte gate) were recorded by flow-cytometry. For thymocyte subset measurement, Alexa-647 conjugated antimouse CD4 (clone: YTS 191) and FITC-conjugated antimouse CD8 (clone: IBL 3/25) antibodies were used (both produced in the Department of Immunology and Biotechnology, University of Pecs, Hungary). For peripheral blood T-cell subpopulation analysis, Pacific Blue-conjugated antimouse CD3 (clone: 17A2), PerCP-conjugated antimouse CD4 (clone: GK1.5), APC/Cy7-conjugated antimouse CD8 (clone: YTS156.7.7), PE-conjugated antimouse CD44 (clone: IM7), APC-conjugated antimouse CD62L (clone: MEL-14) (all purchased form BioLegend), and FITC-conjugated antimouse CD19 (clone: 1D3, produced by the Department of Immunology and Biotechnology, University of Pecs, Hungary) were used.

### T-Cell Recombination Excision Circle (TREC) Measurement by Digital qPCR in Mouse and Human

T-cell recombination excision circle by-products of gene-rearrangement in fresh naive T-cells were also assessed. We performed mouse Trec (mTrec) digital qPCR using mouse and human Trec (hTrec) digital qPCR using human samples by adapting methods published by others (38). Briefly, DNA was isolated from mouse thymocytes using the NucleoSpin Tissue kit (Macherey-Nagel) according to the manufacturer’s instruction. For human, peripheral-blood samples were processed using the DNA Blood Mini kit (Qiagen) following the manufacturer’s guides. Absolute copy numbers were measured by digital PCR on the QuantStudio 3D Digital PCR platform (ThermoFisher) using 30 ng DNA per sample. Taqman primers/probes and digital qPCR reagents were also purchased from ThermoFisher and used as suggested. For age-matched range of healthy human hTrec values, refer to the work of Lynch et al. (38). Permission to perform the described animal experiments was granted to the relevant utilities of the University of Pecs (ref. no.: BA02/2000-46/2016). Experiments involving human blood samples were performed with the consent of the Regional and Local Ethics Committee of Clinical Centre, University or Pecs (ref. no.: 6439/2016) according to their guidelines.

### Oral Tolerance Induction in Mouse

Induction and evaluation of oral tolerance was performed as described by others (39–41). Briefly, both wild-type and PPARgamma haplo-insufficient mice received 5 mg/ml ovalbumin (OVA, Sigma-Aldrich) in drinking water for seven days. On day 7, mice were challenged with an intraperitoneal injection of 5 µg ovalbumin in 200 µl of 1:1 of PBS:complete Freund adjuvant. On day 14, mice received an intraperitoneal injection of 5 µg ovalbumin in 200 µl of 1:1 of PBS:incomplete Freund adjuvant. Serum was collected on day 21 and anti-OVA IgG antibodies were measured by ELISA. Briefly, 96-well Microtest Plates (Sarstedt) were coated with OVA and blocked with BSA. Then plates were incubated with serial dilutions of mouse serum samples (1:100–1:3,200). The antibody content was visualized with the help of HRP-conjugated antimouse immunoglobulin antibody (rabbit polyclonal, Dako). Optical density was measured at 492 nm with iEMS Reader MF equipment (Thermo Labsystems).

### Influenza Vaccination in Mouse

The efficiency of influenza vaccination was investigated as described elsewhere (42). Briefly, both wild-type and PPARgamma haplo-insufficient mice were injected intramuscular once with 0.1 ml human seasonal influenza vaccine cocktail (3Fluart) to mimic human vaccination at 9 months of age. In order to imitate human exposure pattern serum antibody IgG titer against H1N1 A/California/7/2009 strain (part of 3Fluart) was measured by ELISA three months after initial single vaccination at 12 months of age. For detection, ELISA plates were coated with 0.05 μg HA protein of influenza strain A (Recombinant subtype H1N1 A/California/7/2009 His Tag, Life Technologies). Then plates were incubated with serial dilutions of mouse serum samples (1:5–1:1,600). The antibody content was visualized with the help of HRP conjugated a-mouse immunoglobulin antibody (rabbit polyclonal, Dako). Optical density was measured at 492 nm with iEMS Reader MF equipment (Thermo Labsystems).

### Statistical Analysis

All experiments were performed at least on three occasions, representative experiments are shown. Measures were obtained in triplicates, data are presented as mean and +SD as error bars. Graphpad Prism software was used for statistical analysis. Two-tailed Student’s *t*-test was applied. Significant differences are shown by asterisks (ns for not significant, * for *p* ≤ 0.05, ** for *p* ≤ 0.01, *** for *p* ≤ 0.001).

## Results

### PPARgamma Distorts the Ratio of Thymic Epithelial Compartments with Age

Previously reported mouse results showed increasing PPARgamma expression with age in the thymic epithelial compartments, accompanied by thymic adipose involution. We have set out to prove human relevance of previous mouse findings and test whether PPARgamma activity influences the ratio of thymic epithelial compartments.

#### PPARgamma Expression Increases in the Adult Thymus with Age

**Figure 1:**
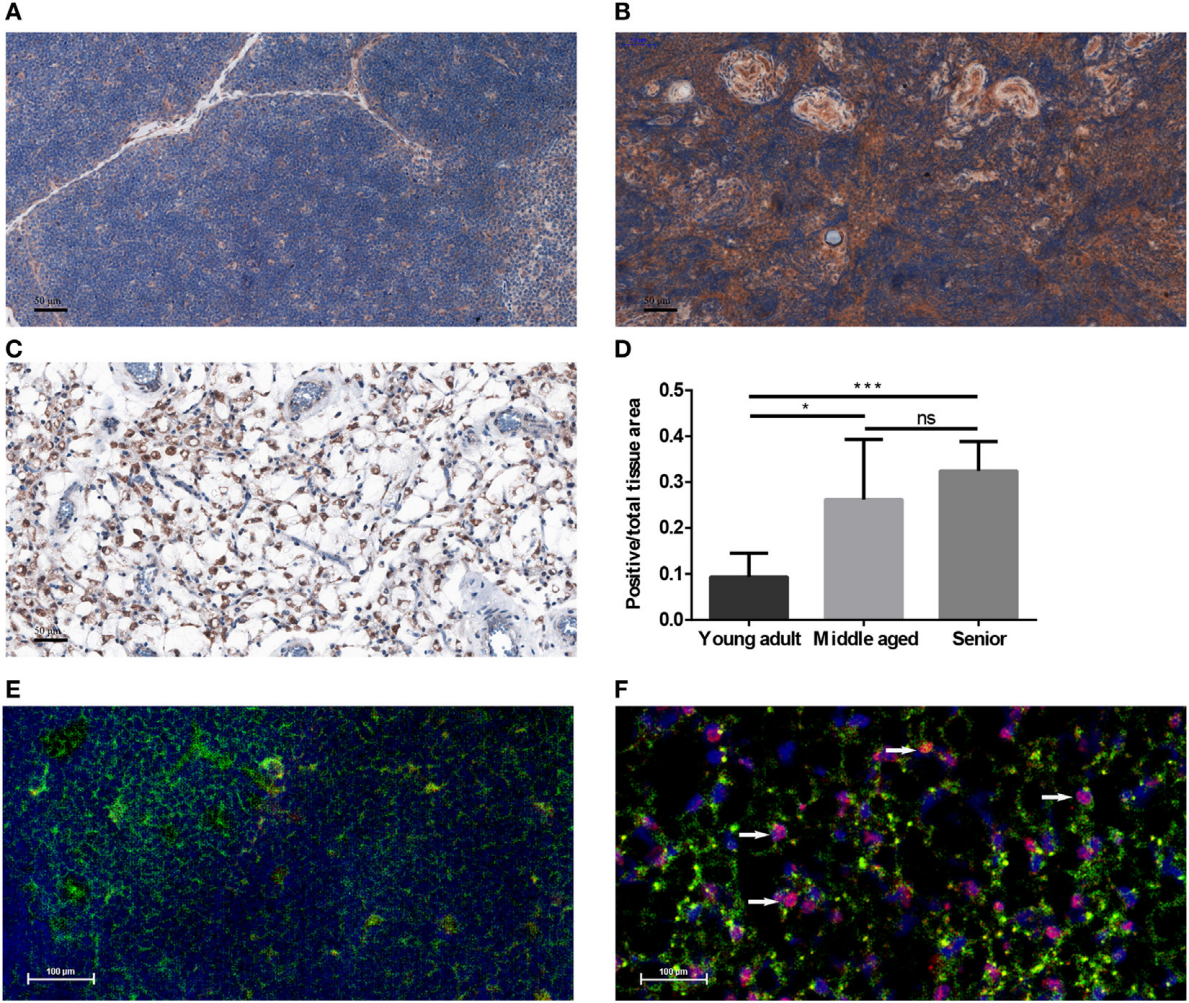
PPARgamma expression in the adult thymus. Human formalin-fixed, paraffin-embedded (FFPE) thymic sections were analyzed for PPARgamma expression by immunohistochemistry in age groups of 20–30 years called young adult **(A)**, 50–60 years called middle-aged **(B)**, and 70–80 years called senior **(C)**. Brown color reaction (DAB) shows PPARgamma expression. Blue color (hematoxylin) shows nuclear counter-stain and defines total cellular areas. The ratio of PPARgamma-expressing cellular areas and total cellular areas is also shown for the different age groups **(D)**. Immunofluorescent staining is also shown for mouse at 1 month of age called young adult and at 15 months of age called senior **(E,F)**. Green color shows epithelial cells (anti-EpCAM1-FITC), red color shows preadipocytes (anti-PPARgamma primary AB with Alexa-555 secondary AB) and blue color defines nuclei (DAPI counter-stain). Note arrowheads pointing at double-staining (EpCAM-1^+^/PPARgamma^+^) cells **(F)**. Both stainings show expected patterns: EpCAM-1 staining presents cell surface markers, while PPARgamma-staining shows nuclear localization (observed in magenta color due to overlap with DAPI nuclear counterstain on Figure 1F). For exact numerical data, refer to Supplementary Material. Significant differences are shown by asterisks (ns for not significant, * for *p* ≤ 0.05, ** for *p* ≤ 0.01, *** for *p* ≤ 0.001).

Human FFPE thymic sections were analyzed for their PPARgamma expression in several adult age groups from young through middle-aged to senior (Figures 1A–D). Our results indicate that PPARgamma expression significantly and progressively increases with age (Figures 1A–C). Of note, total cellular areas shrink at senior ages in both human (Figure 1C) and mouse (Figure 1F). As a result the ratio of PPARgamma-expressing cellular areas shows relative increase with age (Figure 1D). Immunofluorescent staining of mouse thymic cryosections at 15 months of age (Figure 1F) provides visual support for thymic epithelial to adipose transdifferentiation in harmony with the working hypothesis of cellular transdifferentiation. A portion of stromal cells shows dual staining for epithelial identity and adipose differentiation, a hallmark of thymic adipose involution. This phenomenon is not observed at young adult age (Figure 1E).

#### PPARgamma Skews the Ratio of Epithelial Compartments with Age

**Figure 2:**
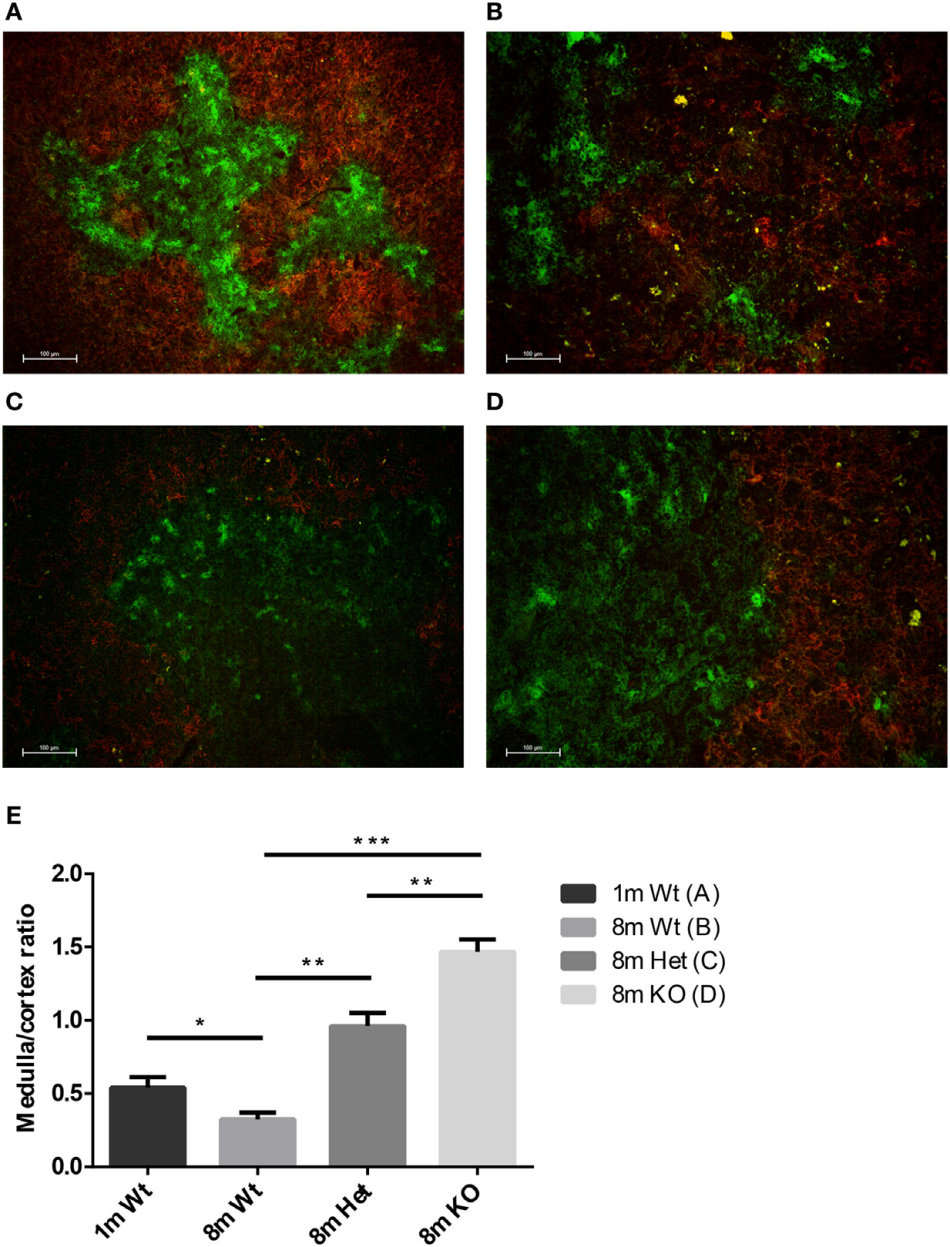
Ratio of epithelial compartments in the adult thymus. Mouse thymic cryosections were stained differentially for medullary (anti-EpCAM1-FITC^++^, anti-Ly51-PE^−^) and cortical (anti-Ly51-PE^++^, anti-EpCAM1-FITC^+^) epithelial compartments. Wild-type thymus is shown at 1 month **(A)** and 8 months of age **(B)**. PPARgamma heterozygous **(C)** and PPARgamma KO **(D)** animals are shown at 8 months of age. The ratio of medullary and cortical epithelial compartment is also shown **(E)** for both ages and genetic backgrounds. For exact numerical data, refer to Supplementary Material. Significant differences are shown by asterisks (ns for not significant, * for *p* ≤ 0.05, ** for *p* ≤ 0.01, *** for *p* ≤ 0.001).

Mouse thymic cryosections were differentially stained for medullary and cortical epithelial compartments at several ages and using various genetic backgrounds (Figures 2A–D). Our results show that in the wild-type setting the medullary epithelial compartment significantly shrinks with age as reported previously (31). This, however, is not observed in PPARgamma deficient settings. Loss of PPARgamma activity shows protection in a progressive manner presenting dose–response (Figure 2E). PPARgamma deficiency efficiently and significantly prevents the erosion of the medullary epithelial compartment, otherwise prone to shrink with senescence.

### PPARgamma Affects Thymic T-Cell Production and Peripheral Blood T-Cell Distribution with Age

We have observed changes in thymus architecture in response to PPARgamma status. Consequently, we were interested in whether morphological changes alter thymus function: naive T-cell production. Going beyond, we were eager to see if sustained influence of PPARgamma status on thymocyte function is also reflected in the peripheral blood.

#### PPARgamma Disturbs Thymic T-Cell Output with Age

**Figure 3:**
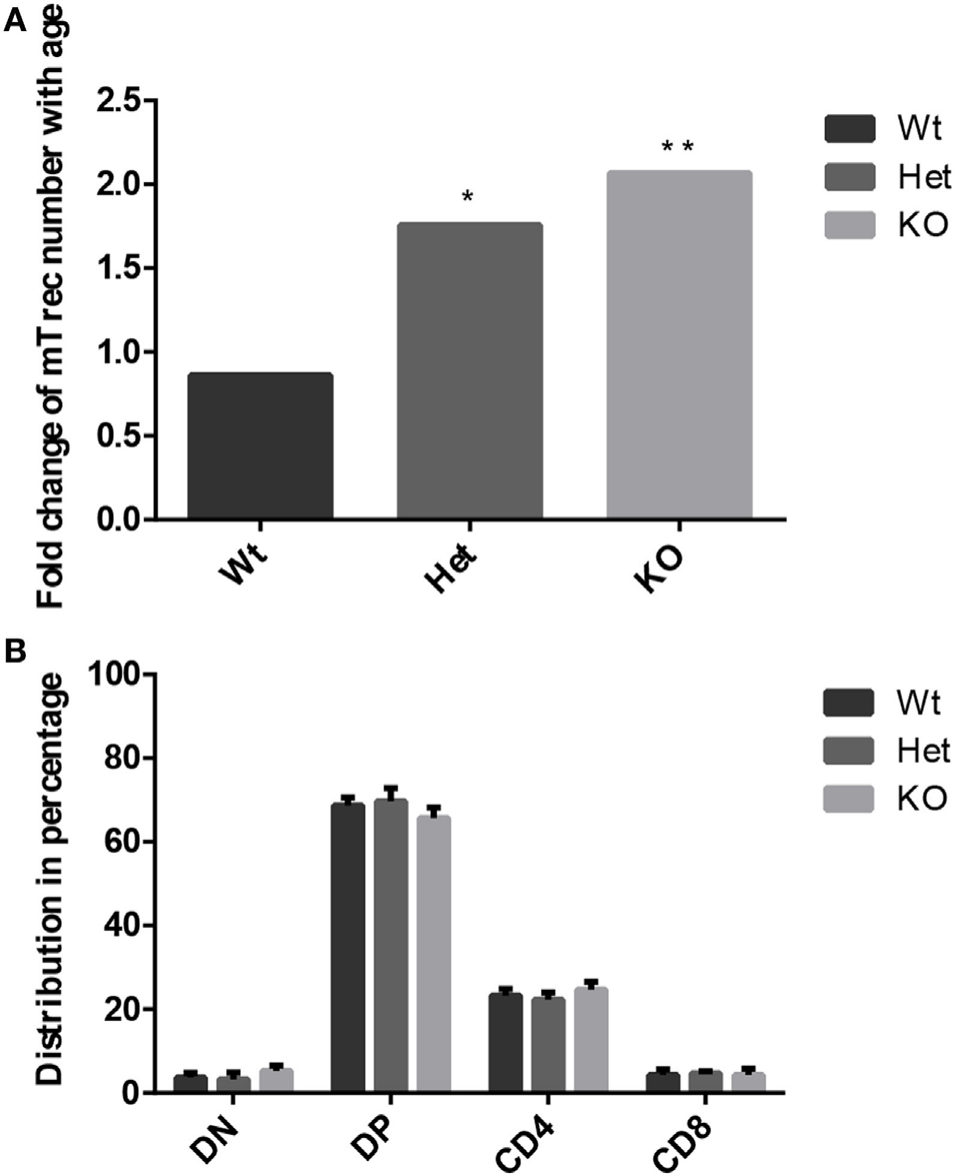
Thymocyte development in the adult thymus. Changes in level of mouse T-cell recombination excision circles (mTrecs) was evaluated by Taqman digital qPCR in wild-type, PPARgamma heterozygous, and PPARgamma KO thymocytes **(A)**. The columns represent mTrec values measured at 8 months divided by those measured at 1 month for every strain. The ratio of thymocyte subpopulations was assessed by flow-cytometry at 8 months of age in wild-type, PPARgamma heterozygous and PPARgamma KO animals **(B)**. Double negative (CD4^−^, CD8^−^), double positive (CD4^+^, CD8^+^), and single positive (CD4^+^ or CD8^+^) subpopulations are shown. For the measurement of every sample, 100,000 cells were stained and 10,000 events (parent R1 morphological lymphocyte gate) were recorded by flow-cytometry. For exact cell numbers, refer to Supplementary Material. Significant differences are shown by asterisks (ns for not significant, * for *p* ≤ 0.05, ** for *p* ≤ 0.01, *** for *p* ≤ 0.001).

Age-related changes in thymocyte levels of mTrec (DNA loop by-product of mouse T-cell receptor gene rearrangement) were evaluated in wild-type and PPARgamma deficient settings using digital qPCR (Figure 3A). Our results indicate slight (though not significant) decrease of mTrec and hence fresh-naive T-cell output with age in thymocytes of wild-type mice. PPARgamma deficiency significantly and progressively counteracts the process also showing dose-responsive increase of thymocyte mTrec levels. In further analyses, the percent distribution of thymocyte subpopulations was assessed using flow-cytometry in wild-type and PPARgamma deficient mice (Figure 3B). All thymocyte subpopulations showed near identical distribution pattern with all genetic backgrounds. Taken together, PPARgamma deficiency progressively enhances thymocyte development in adult age, but without skewing the distribution of thymocyte subpopulations or their differentiation preference.

#### PPARgamma Influences T-Cell Subpopulation Distribution in Adult Peripheral Blood

**Figure 4:**
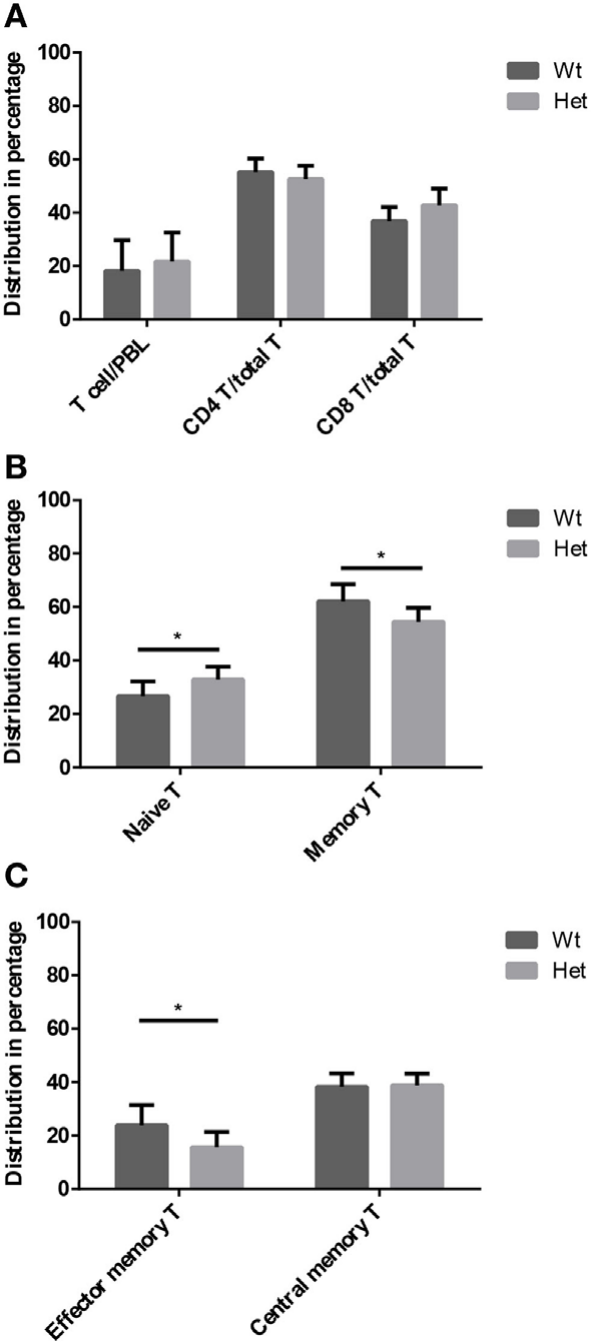
T-cell subpopulations in adult peripheral blood. Peripheral blood T-cell subpopulations were evaluated by flow-cytometry at 12 months of age in wild-type and PPARgamma heterozygous animals (KO animals decease by this age). Percent distribution of T-cells (CD3^+^), helper T-cells (CD3^+^, CD4^+^), and cytotoxic T-cells (CD3^+^, CD8^+^) is shown by **(A)**. Also, the percent distribution of naive T-cells (CD3^+^, CD44^−^, CD62L^+^) and memory T-cells (CD3^+^, CD44^+^, CD62L^+/−^) was evaluated within the CD3-gate of T-cells **(B)**. Further analysis of memory T-cell subpopulation shows percent distribution of effector memory T-cells (CD3^+^, CD44^+^, CD62L^−^) and central memory T-cells (CD3^+^, CD44^+^, CD62L^+^) within the CD3-gate of T-cells **(C)**. For the measurement of every sample, 100,000 cells were stained and 10,000 events (parent R1 morphological lymphocyte gate) were recorded by flow-cytometry. For exact cell numbers, refer to Supplementary Material. Significant differences are shown by asterisks (ns for not significant, * for *p* ≤ 0.05, ** for *p* ≤ 0.01, *** for *p* ≤ 0.001).

Peripheral blood T-cell subpopulations were evaluated by flow-cytometry at 12 months of age in wild-type and PPARgamma deficient animals. Our results do not show differences in the percent distribution of the major T-cell groups of helper T-cells and cytotoxic T-cells (Figure 4A) within the CD3-gate of T-cells. However, the evaluation of naive T-cell and memory T-cell ratio reveals significant effect of PPARgamma deficiency (Figure 4B). There is significant increase of naive T-cells in the peripheral blood of PPARgamma deficient animals compared to wild-type animals, conversely and significantly decreasing the memory T-cell pool within the CD3-gate of T-cells. Deeper analysis of the memory T-cell pool reveals it is the mobile effector memory T-cell subpopulation that shows significant decrease and not central memory T-cells (Figure 4C) within the CD3-gate of T-cells. Sustained and prolonged naive T-cell production due to PPARgamma deficiency in the thymus as suggested by mTrec values above apparently affects peripheral blood T-cell subpopulations as shown here.

### Functional Immunological Consequence and Human Relevance

Having seen the far-reaching influence of PPARgamma status on thymus architecture, thymus function and peripheral blood T-cell composition with age, we have set out to test whether these changes have functional immunological relevance. If so, it would be also of high interest to test if our comprehensive mouse results have human relevance.

#### PPARgamma Modulates Immune Regulation and Immune Response

**Figure 5:**
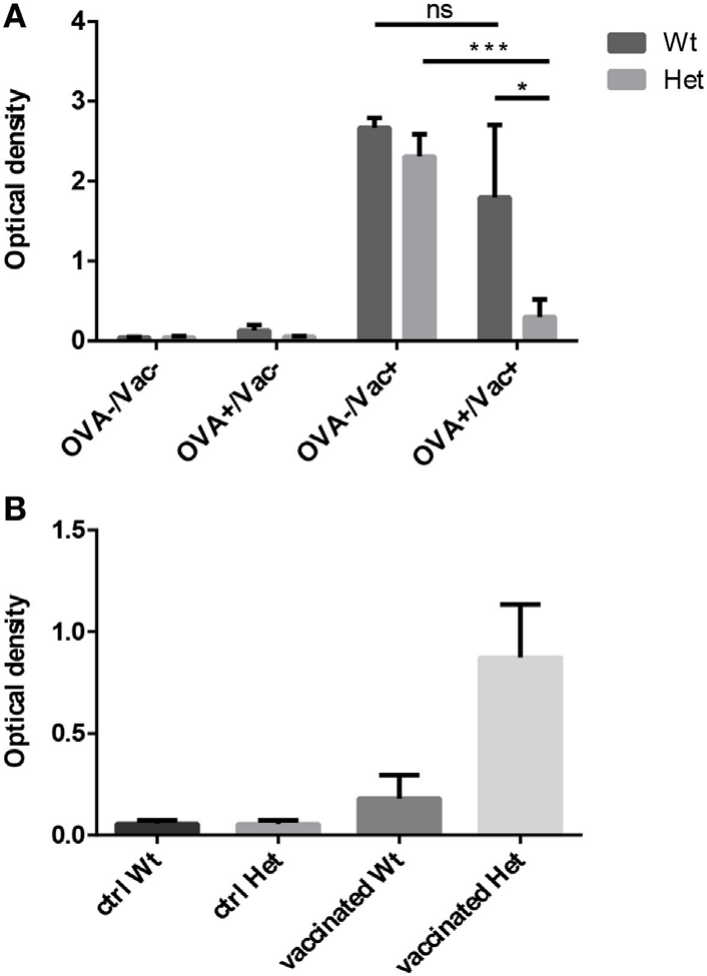
Functional immunological experiments in adult hosts. Oral tolerance induction capacity to ovalbumin (OVA) was assayed in wild-type and PPARgamma heterozygous animals at 12 months of age. Animals received OVA by either drinking water, i.p. injection, both or neither. OVA-specific IgG titers were evaluated 3 weeks later by ELISA method **(A)**. The presented figure was obtained using 1:400 dilution of serum. Mean ELISA OD values are shown for each study group. Human seasonal influenza vaccine (3Fluart) was injected (0.1 ml, 1×, i.m.) into wild-type and PPARgamma heterozygous animals at 9 months of age. Serum IgG titers specific to a vaccine component (H1N1 A/California/7/2009 strain) were tested 3 months later by ELISA method **(B)**. The presented figure was obtained using 1:50 dilution of serum. Maximal ELISA OD values are shown for each study group. For exact numerical data, refer to Supplementary Material. Significant differences are shown by asterisks (ns for not significant, * for *p* ≤ 0.05, ** for *p* ≤ 0.01, *** for *p* ≤ 0.001).

We have tested the capacity to mount oral tolerance to the foreign protein OVA in wild-type and PPARgamma deficient aged adult mice by measuring OVA-specific IgG titers following oral and/or intraperitoneal OVA challenge (Figure 5A). As reported by others, age impairs oral tolerance in wild-type animals (40, 41). As a consequence, there is only moderate, insufficient decrease of OVA-specific IgG titers in case of parallel oral OVA administration and i.p. OVA-injection in senior animals. However, PPARgamma deficiency rescues oral tolerance in the same experimental setting despite age, profoundly and significantly decreasing OVA-specific IgG titers (Figure 5A). Consequently, naive T-cell dependent immune regulation (oral tolerance) remains efficient in PPARgamma heterozygous animals despite their age.

The capacity to mount immune reaction to foreign influenza antigens was also tested as human seasonal influenza vaccine was injected into aged adult wild-type and PPARgamma deficient animals. Subsequent analysis of serum IgG titers specific to a vaccine component showed elevated protective antibody production (maximal ELISA OD values) in PPARgamma deficient animals, but not in their wild-type littermates (Figure 5B). This tendency is not significant because of individual variation observed due to the applied human vaccination protocol being inferior to standard mouse immunization protocol. Nevertheless, naive T-cell dependent immune response proves to be efficient in aged, PPARgamma heterozygous animals.

#### Human Evidence of PPARgamma Deficiency Preventing Thymic Senescence

**Figure 6:**
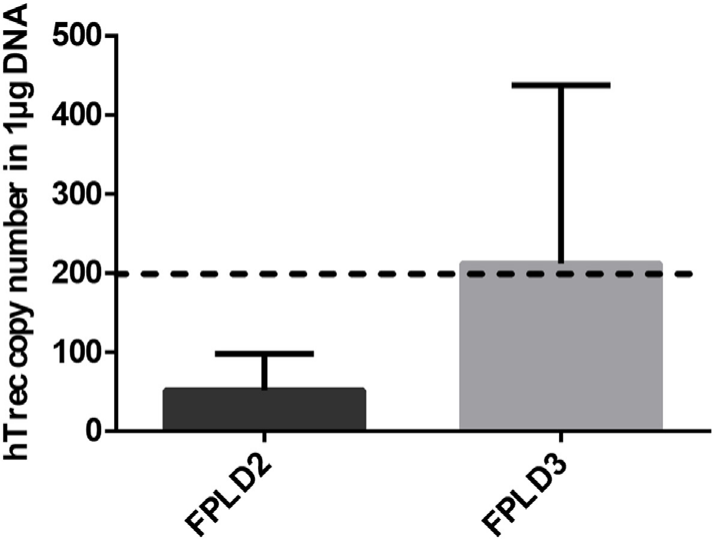
Thymus function in adult FPLD patients. Level of human T-cell recombination excision circle (hTrec) was measured by Taqman digital qPCR in peripheral blood leukocytes of age-matched and disease-matched rare disease patients with FPLD2 condition (lipodystrophy due to LMNA deficiency) and FPLD3 condition (lipodystrophy due to PPARgamma deficiency) (Figure 6). Patient sample numbers were *n* = 3 for FPLD2 and *n* = 5 for FPLD3. For exact numerical data, refer to Supplementary Material. For age-matched (approx. 50 years of age) range of healthy human hTrec values, refer to the work of Lynch et al. (38). Accordingly, the lower limit of healthy human hTrec threshold (approximaterly 200 copies/μg DNA) is represented by dotted line.

Genetic PPARgamma deficiency is a rare, but existing condition in human called FPLD3 (15). It leads to a metabolic phenotype called lipodystrophy, similar to the mouse (11–15). Other rare human conditions not affecting PPARgamma can also lead to lipodystrophy (12–15). In case of FPLD2 lamin mutations trigger similar metabolic changes (14). Peripheral blood hTrec (DNA loop by-product of human T-cell receptor gene rearrangement) levels were measured using digital qPCR in age-matched patients with FPLD2 condition and FPLD3 condition (Figure 6). As expected and in perfect harmony with previous mouse thymocyte results elevated mean hTrec levels were detected in FPLD3 samples compared to FPLD2 samples. The tendency is not significant due to individual variation within the patient groups. Unfortunately, current patient sample numbers cannot be increased due to the extremely rare nature of these conditions (FPLD2 or ORPHA 2348 has prevalence of ≤1/1,000,000 and FPLD3 or ORPHA 79083 also has prevalence of ≤1/1,000,000) (14, 15). For age-matched range of healthy human hTrec values, refer to the work of Lynch et al. (38). Lower limit of healthy human hTrec threshold (approx. 200 copies/μg DNA) is not reached by FPLD2 (lamin) patient samples, but this is rescued in FPLD3 (PPARgamma) patients despite being age and disease matched.

## Discussion

### PPARgamma Drives Thymic Epithelial to Adipose Trans-Differentiation with Age

It has been previously suggested based on direct fate-mapping experiments that with senescence thymic adipose tissue develops from the thymic stromal or epithelial compartment (28). Based on indirect evidence others have also supported this concept (29). In further support, we here present visual evidence of epithelial to adipose transdifferentiation in the mouse. This is indicated by the presence by EpCAM-1/PPARgamma double-positive cells shown by histology (Figure 1D). These cells still express cell surface markers of their fading thymic epithelial identity (EpCAM-1), but already show early signs of the novel adipocyte differentiation program in their nuclei (PPARgamma). The fact that such double positive cells show rather scattered and not uniform staining pattern at a given time point may provide explanation for gradual thymic adipose involution observed during senescence.

### PPARgamma Impairs Naive T-Cell Production with Age

Thymus histology data show that the medullary compartment is rescued from age-related shrinking in case of PPARgamma deficiency (Figures 2A–D). Extended survival of this stromal niche ensures permissive environment for sustained thymus function: naive T-cell production. This is indicated by elevated mTrec values showing direct correlation with PPARgamma deficiency (Figure 3A). Of extreme importance and highlighting human relevance, peripheral blood leukocyte hTrec values from adult FPLD3 patients (with genetic PPARgamma deficiency) also exceed adult FPLD2 patient values (with unrelated genetic background) despite being age-matched and disease-matched (lipodystrophy, diabetes) (Figure 6). Of note, such metabolic disorders are known to impair thymus function indicated by decreased hTrec values as reported by others (43, 44). For exactly, this reason have we used disease-matched controls (FPLD2 vs. FPLD3) to show enhanced thymus function with PPARgamma deficiency despite metabolic disorders. Unlike lower than physiological hTrec values measured in FPLD2 (lamin) patients, those measured in FPLD3 (PPARgamma) patients are within healthy human physiological range (Figure 6). Since both mTrec and hTrec DNA loops originate from gene rearrangement during thymocte development this is direct evidence of sustained T-cell development indicating intact thymic niche in PPARgamma deficient animal models and human patients (38). Of note, the distribution of thymocyte subpopulations shows identical pattern irrespective of PPARgamma status proving that sustained, enhanced thymocyte development does not skew differentiation preference, but rather enhances fresh, naive T-cell production of all thymocyte subtypes uniformly (Figure 3B). Finally, since sustained thymic naive T-cell production is not restricted to a given time-point, but rather represents a continuous trend, the peripheral blood naive T-cell population shows cumulative differences as it is rescued from age-driven shrinking, against the memory T-cell population—more specifically against the effector memory T-cell pool (Figures 4B,C).

### PPARgamma Hampers T-Dependent Immune Regulation and Immunity with Age

Oral consumption of foreign T-depended antigen normally initiates immune tolerance inhibiting any eliminative immune response (e.g., serum IgG), despite parallel immunization in young adult individuals with appropriate naive T-cell supply. Unfortunately, the phenomenon is disrupted at senior age due to the lacking naive T-cell pool in the Peyer’s patches of the gut (40, 41, 45) This loss of oral tolerance (impaired immune regulation) is a possible link to increasing food intolerance prevalence observed in the aging adult population (46–49). However, the phenomenon may be rescued by PPARgamma deficiency despite age providing evidence that sustained T-cell production is necessary for efficient oral (immune) tolerance (Figure 5A).

Senescence-triggered decrease of naive T-cell output also impairs T-dependent immunity. An example in the senior human population is decreased protection from seasonal flu strains despite annual vaccination campaigns (50–52). The phenomenon has well established animal models (53–55). This is caused by low levels of neutralizing antibody titers due to lacking naive T-cells necessary during T-B cooperation to mount adequate innate immune response against T-dependent antigens of the vaccine. This, however, is not the case with PPARgamma deficiency (Figure 5B). Single intramuscular vaccination against seasonal flu (mimicking human vaccination campaign) resulted in higher maximal antibody production three months later (a typical delay in human exposure). This confirms that the cause of decreased vaccination efficiency in the senior population is impaired T-dependent immunity due to thymic senescence.

In our experiments, we have focused on the decline of T-dependent immunity since the thymus shows early and dramatic signs of senescence during adipose involution. This, however, is not the case for the B-cell compartment for which aging has been reported to occur later and in a more gradual fashion, lacking such profound histological changes (56).

PPARgamma is an enigmatic transcription factor showing unique expression pattern in both time and space throughout the body (57). PPARgamma affects both hemopoietic and stromal compartments during development and aging. Further dissection would require to perform, e.g., bone-marrow transplantation experiments between control and PPARgamma deficient animals. However, PPARgamma KO animals develop severe metabolic disorders that hamper such experiments, especially at elevated ages.

### Limitations and Perspectives

We here present the long-term thymus- and T-dependent immunity-preserving effect of systemic (genetic) loss of PPARgamma function as observed in PPARgamma deficient mouse models and in a human rare disease (FPLD3). In both cases, there are severe metabolic drawbacks (diabetes, dyslipidemia etc.) due to systemically lacking PPARgamma activity. However, alternative, thymus tissue-restricted suppression of PPARgamma activity would likely solve the issue. Of note, as reported previously, overexpression of Wnt4 glycolipoproteins by thymic epithelial cells can efficiently counteract PPARgamma (31). Also, Wnt4 was described to travel in extracellular vesicles including exosomes and affect thymocyte differentiation (58, 59). Hence, it is conceivable that thymic epithelium-derived, enriched exosomes would efficiently home to the thymus and deliver their Wnt4 cargo locally even when administered systemically. This would, in theory, allow for the natural, tissue-specific, protein-mediated maintenance of thymic epithelial identity and prevent thymic senescence from developing.

Although tissue senescence is ultimately inevitable, there are conditions that accelerate thymic senescence including certain viral infections, intoxications, irradiation, chemotherapy, etc. Outcomes include increased incidence of infection, cancer and autoimmune disorder. In any case, the identification of molecular level targets for potential intervention is highly desired. Therefore, molecular level insight into immune senescence has medical, economical, and personal relevance, all at once.

## Ethics Statement

Experiments involving human thymus samples were performed with the consent of the Regional and Local Ethics Committee of Clinical Centre, University or Pecs (ref. no.: 6331/2016) according to their guidelines. Experiments involving human blood samples were performed with the consent of the Regional and Local Ethics Committee of Clinical Centre, University or Pecs (ref. no.: 6439/2016) according to their guidelines. All subjects gave written informed consent in accordance with the Declaration of Helsinki. Permission to perform the described animal experiments was granted to the relevant utilities of the University of Pecs (ref. no.: BA02/2000-46/2016). Permission to generate PPARgamma GM mice was granted to the relevant utilities of the University of Debrecen (ref. no.: TMF/82-10/2015). Permission to perform experimental procedures with PPARgamma GM mice was granted to the relevant utilities of the University of Pecs (ref. no.: TMF/124-11/2017).

## Author Contributions

DE performed most histological, molecular biology, and statistics work in the project and was involved in manuscript preparation. KB performed all human IHC work. ZK performed oral immune tolerance experiments. AP was in charge for the breeding, metabolic, and genetic characterization of PPARgamma haplo-insufficient and null mice. JL was in charge for planning human experiments, involved in manuscript preparation as well as local supervision of respective department. PE was involved in planning mouse experiments, involved in manuscript preparation as well as local supervision of respective department. KK was involved in histological, molecular biology and statistics work, also in planning experiments and manuscript preparation, and supervised the project.

## Conflict of Interest Statement

The authors declare that they have no conflicts of interest with the contents of this article. The research was conducted in the absence of any commercial or financial relationship that could be construed as a potential conflict of interest.
