# Supplementary material for: PPARgamma Deficiency Counteracts Thymic Senescence
Source: Front Immunol. 2017 Nov 6;8:1515. doi: 10.3389/fimmu.2017.01515 (PMC5681731; doi:10.3389/fimmu.2017.01515)
Supplement: Supplementary file 1 [file Data_Sheet_1.DOCX]

**SUPPLEMENTARY MATERIAL**

**Numerical values of figures**

**Figure 1E.**

Ratio of PPARgamma-stained cellular area divided by total cellular area on human thymus sections

| **Age group** | **Mean** | **SD** |
| --- | --- | --- |
| 20-30 years | 0.093 | 0.052 |
| 50-60 years | 0.262 | 0.130 |
| 70-80 years | 0.324 | 0.064 |

**Figure 2.**

Ratio of medullary area divided by cortical area on mouse thymus sections

| **Age group / background** | **Mean** | **SD** |
| --- | --- | --- |
| 1 month / wild-type | 0.540 | 0.072 |
| 8 months / wild-type | 0.324 | 0.047 |
| 8 months / PPARgamma hetero | 0.962 | 0.089 |
| 8 months / PPARgamma KO | 1.467 | 0.085 |

**Figure 3A**.

Fold change of mTrec level with age in mouse

| **mTrec ratio with age:**  8 months / 1 month | **Wild-type** | **PPARgamma hetero** | **PPARgamma KO** |
| --- | --- | --- | --- |
|  | 0.862 | 1.757 | 2.067 |

**Figure 3B**.

Distribution of thymocyte subpopulations in mouse

(percent of parent R1 morphological lymphocyte gate and event count)

|  | **Wild-type** | | **PPARgamma hetero** | | **PPARgamma KO** | |
| --- | --- | --- | --- | --- | --- | --- |
|  | **Mean** | **SD** | **Mean** | **SD** | **Mean** | **SD** |
| DN  CD4^-^ CD8^-^ | 3.67% R1  3667 events | 1.16% | 3.33%  3333 events | 1.53% | 5.33%  5333 events | 1.18% |
| DP  CD4^+^ CD8^+^ | 68.67% R1  6867 events | 1.95% | 69.67%  6967 events | 3.22% | 65.67%  6567 events | 2.51% |
| SP  CD4^+^ CD8^-^ | 23.33% R1  2333 events | 1.53% | 22.33%  2233 events | 1.71% | 24.67%  2467 events | 1.86% |
| SP  CD4^-^ CD8^+^ | 4.33% R1  4333 events | 1.27% | 4.67%  4667 events | 0.58% | 4.33%  4333 events | 1.44% |

**Figure 4A.**

Distribution of T-cell population and subpopulations in peripheral blood in mouse

(percent of parent R1 morphological lymphocyte gate or parent CD3 T-cell gate and event count)

|  | **Wild-type** | | **PPARgamma hetero** | |
| --- | --- | --- | --- | --- |
|  | **Mean** | **SD** | **Mean** | **SD** |
| T-cells / PBL | 18.21% R1  1821 events | 11.51% | 21.67%  2167 events | 10.89% |
| CD4^+^ / T-cells | 55.18% CD3  1005 events | 5.14% | 52.66%  1141 events | 4.99% |
| CD8^+^ / T-cells | 36.90% CD3  672 events | 5.22% | 42.76%  927 events | 6.28% |

**Figure 4B.**

Distribution of T-cell subpopulations within T-cells in peripheral blood in mouse

(percent of parent CD3 T-cell gate and event count)

|  | **Wild-type** | | **PPARgamma hetero** | |
| --- | --- | --- | --- | --- |
|  | **Mean** | **SD** | **Mean** | **SD** |
| Naïve T-cells (CD62L^+^) | 26.69% CD3  486 events | 5.43% | 32.90%  713 events | 4.72% |
| Memory T-cells (CD44^+^) | 62.13% CD3  1131 events | 6.41% | 54.48%  1181 events | 5.22% |

**Figure 4C.**

Distribution of T-cell subpopulations within T-cells in peripheral blood in mouse

(percent of parent CD3 T-cell gate and event count)

|  | **Wild-type** | | **PPARgamma hetero** | |
| --- | --- | --- | --- | --- |
|  | **Mean** | **SD** | **Mean** | **SD** |
| Effector memory T (CD44^+^ CD62L^-^) | 23.85% CD3  434 events | 7.61% | 15.59%  338 events | 5.77% |
| Central memory T (CD44^+^ CD62L^+^) | 38.28% CD3  697 events | 5.02% | 38.89%  843 events | 4.29% |

**Figure 5A.**

Oral intolerance experiment using ovalbumin (OVA) in mouse (average ELISA OD values)

| **OVA**  **oral / injected** | **Wild-type** | | **PPARgamma hetero** | |
| --- | --- | --- | --- | --- |
|  | **Mean** | **SD** | **Mean** | **SD** |
| **- / -** | 0.041 | 0.009 | 0.043 | 0.016 |
| **+ / -** | 0.127 | 0.071 | 0.050 | 0.008 |
| **- / +** | 2.669 | 0.123 | 2.307 | 0.280 |
| **+ / +** | 1.794 | 0.910 | 0.297 | 0.220 |

**Figure 5B.**

Immunization challenge using influenza vaccine (3Fluart) in mouse (maximal ELISA OD values)

| **Wild-type / ctrl** | | **PPARg hetero / ctrl** | | **Wild-type / vacc.** | | **PPARg hetero / vacc.** | |
| --- | --- | --- | --- | --- | --- | --- | --- |
| **Mean** | **SD** | **Mean** | **SD** | **Mean** | **SD** | **Mean** | **SD** |
| 0.055 | 0.017 | 0.054 | 0.019 | 0.181 | 0.115 | 0.874 | 0.261 |

**Figure 6.**

Naïve T-cell production in human FPLD patients of different genetic background (hTrec qPCR)

| **FPLD2** | | **FPLD3** | |
| --- | --- | --- | --- |
| **Mean** | **SD** | **Mean** | **SD** |
| 51.67 | 46.32 | 212.23 | 225.6 |
